# Spatiotemporal expression pattern of dyslexia susceptibility 1 candidate 1 (DYX1C1) during rat cerebral cortex development

**DOI:** 10.1038/s41390-025-03920-6

**Published:** 2025-02-12

**Authors:** Kazumasa Zensho, Ikuko Miyazaki, Aika Isse, Ichika Misawa, Kaori Masai, Makio Oka, Hirokazu Tsukahara, Masato Asanuma

**Affiliations:** 1 Department of Medical Neurobiology, Okayama University Graduate School of Medicine, Dentistry and Pharmaceutical Sciences, Okayama, 700-8558 Japan; 2 Department of Pediatrics, Okayama University Hospital, Okayama, 700-8558 Japan; 3 Department of Pediatrics, Kurashiki Medical Center, Okayama, 710-8522 Japan; 4 Department of Psychosocial Medicine, National Center for Child Health and Development, Tokyo, 157-8535 Japan

## Abstract

**Background:**

Developmental dyslexia (DD) is a common learning disorder with significant consequences for affected individuals. Although several candidate genes, including dyslexia susceptibility 1 candidate 1 (DYX1C1), have been implicated in dyslexia, their role in brain development remains unclear. We aimed to elucidate the spatiotemporal expression patterns of DYX1C1 during cerebral cortex development in rats.

**Methods:**

We investigated DYX1C1 expression during cerebral cortex development using rat embryos at various gestational stages (E13.5, 15.5, 17.5 and 20.5) by immunohistochemistry (*n* = 7 embryos/stage), quantitative real-time PCR (*n* = 6), and in situ hybridization (*n* = 11–15).

**Results:**

The DYX1C1-positive cells were predominantly located in the outermost layers of the cortical plate, particularly at E15.5. DYX1C1 mRNA expression peaked at E15.5 and subsequently declined. DYX1C1-positive cells did not co-localize with reelin-positive Cajal-Retzius cells, but co-localized with neuronal markers expressed during development, and had shorter primary cilia than DYX1C1-negative cells.

**Conclusions:**

Our findings highlight the dynamic expression of DYX1C1 in the developing cerebral cortex of rats, implicating its involvement in neurodevelopmental processes. Further investigation of the functional interactions of DYX1C1, particularly its relationship with reelin and its role in cerebrocortical and hippocampal development, may provide insights into the pathophysiology of dyslexia and neurodevelopmental disorders.

**Impact:**

- Our study elucidates spatiotemporal expression patterns of endogenous DYX1C1 predominantly in the primitive cortical zone (PCZ), outermost layer of the cortical plate (CP) during cerebral cortex development, particularly peaked at E15.5.
- We revealed the spatial relationship between DYX1C1-positive and reelin-expressing Cajal-Retzius (CR) cells, and co-localize with neuronal markers expressed during cerebral cortex development, indicating its contribution to neuronal migration and cortical layer formation.
- DYX1C1-positive cells mainly in the PCZ possess shorter primary cilia than DYX1C1-negative cells, suggesting the completion of migration.

## Introduction

Developmental dyslexia (DD) is one of the most prevalent neurodevelopmental disorders, affecting approximately 5–10% of elementary school students worldwide.^1^ Affected individuals presents with difficulties in reading accurately and fluently, despite having normal intelligence and no neurological abnormalities, and face challenges in decoding and spelling words.^2^ DD has a significant secondary impact on mental health, increasing the risk of anxiety disorders in DD by 3.80-fold compared to the general prevalence of 9.4% in children aged 3–17 years (National Institute of Mental Health, 2016–2019), and the risk of depression in DD is increased by 1.99-fold compared to the general prevalence of 4.4% in children aged 3–17 years.^3,4^ Therefore, approximate 36% and 9% of DD shows anxiety and depression, respectively, resulting in the avoidance of school settings. Despite recent advances, there remains a lack of understanding of the pathophysiology and social recognition of dyslexia.^5^ There is an urgent need to elucidate the underlying mechanism and pathophysiology of DD, not only for its prevention and treatment but also for enhancing societal awareness and developing appropriate support systems.^6^

Genetic factors play a pivotal role in the development of DD, and several candidate genes have been implicated in its etiology.^7^ In particular, translocation t(2;15)(q11;q21) and SNPs of dyslexia susceptibility 1 candidate 1 (DYX1C1) gene,^8–10^ intron 2 deletion and SNPs of doublecortin domain containing 2 (DCDC2) gene,^11^ and SNPs of dyslexia-associated protein (KIAA0319) gene have been reported with remarkable interest.^12^ The existing literature on these genetic variations has been subjected to extensive review.^5–7^ Although animals with these gene mutation were used as model of DD, it is hard to evaluate dyslexia in the animals. Therefore, animal study focuses on specific endophenotypes such as neuronal migration, cerebral cortex development, and brain lateralization. Previous studies have used gene suppression method with RNA interference (RNAi) to provide invaluable insights into the underlying mechanisms, particularly regarding disruptions in neuronal migration during cerebral cortex development.^5,13^ Notably, DYX1C1 has emerged as one of the earliest candidate genes associated with DD.^8,9^ This gene encodes a tetratricopeptide repeat (TPR) domain-containing protein.^9^ TPR is a broadly present structural motif that aids in protein-protein interactions and the formation of multi-protein structures. DYX1C1 is also implicated in neuronal migration, ciliary function, and cytoskeletal interactions. Mutations in DYX1C1 are associated with primary ciliary dyskinesia (PCD), highlighting their involvement in conditions beyond dyslexia.^14^ Dysfunctions in ciliary structure and function, termed ciliopathies, are linked to neurodevelopmental phenotypes.^15^ Note that mutations in DCDC2 are associated with hearing impairment due to abnormal cochlear cilia, and there have been no reports yet of symptoms other than DD due to changes in KIAA0319.^5,6^ Understanding its expression during neuronal migration is crucial to elucidating its role in cerebrocortical development. Given previous studies indicating that DYX1C1 knockdown impedes neuronal migration in the developing cerebral cortex,^16–18^ we investigated its spatiotemporal expression pattern at embryonal timepoints. Postnatal analysis would provide insights into its role in brain maturation and function. While previous RNA-based studies have shown widespread DYX1C1 expression, the precise localization of DYX1C1 protein during critical periods of neuronal migration and differentiation remains unclear. Postmortem examination of the brains of individuals diagnosed with DD revealed microscopic abnormalities, predominantly in the left hemisphere.^19,20^ These findings suggest that disrupted neuronal callosal crossing migration is a potential cause of this disorder.^5^ However, the role of DYX1C1, a gene associated with DD, in cerebral cortex development remains unclear, particularly with respect to neurodevelopment.

In this study, we examined the spatiotemporal expression patterns of DYX1C1 during cerebral cortex development in rats, to elucidate its distribution and dynamics in neuronal migration. Understanding these developmental roles may provide mechanistic insights into how DYX1C1 dysregulation leads to the neuropathological and cognitive manifestations of DD.

## Materials and methods

### Animal handling and ethical considerations

All animal procedures adhered to the Okayama University Guidelines for Animal Experiments and were approved by the university’s Committee on Animal Experimentation (approval number: OKU-2022753). Pregnant female Sprague-Dawley rats were procured from Jackson Laboratory Japan, Inc. (Yokohama, Japan). The day following mating was designated as embryonic development day (E) 0, at noon of the following day defined as E 0.5 to account for the approximate time of fertilization. Embryos from pregnant Sprague-Dawley rats at various gestational stage (E13.5, E15.5, E17.5, and E20.5) were used for immunohistochemistry (*n* = 7 embryos per developmental stage), quantitative real-time polymerase chain reaction (PCR) (*n* = 6 embryos/stage), and in situ hybridization histochemistry (*n* = 11~15 embryos/stage), to investigate natural spatiotemporal expression pattern of DYX1C1 at embryonal timepoints, because the previous studies showed that DYX1C1 knockdown inhibited neuronal migration in the developing cerebral cortex.^16–18^ According to the previous reports showing microscopic abnormalities predominantly in the left hemisphere,^19,20^ sections of the left hemisphere were used in the immunostaining. For immunohistochemistry, embryos were euthanized by cervical dislocation, and all heads were promptly immersed in ice-cold 4% paraformaldehyde (PFA) to preserve their anatomical integrity. For histological analysis, the left hemisphere was used for the analyses and the right half was used for just making orientation. Notably, at E17.5 and E20.5, the removal of the cerebral dura mater and developing skull preceded fixation to enhance solution penetration. This step was essential for maintaining staining quality, as indicated by preliminary observations (data not shown). For RNA extraction, embryonic rat cerebral cortex tissue was isolated from E13.5, 15.5, 17.5, 20.5 brains (*n* = 6 embryos/stage) using previously established methods.^21^

### Immunohistochemistry

Embryonic rat brains (E13.5–E20.5, *n* = 7 embryos per developmental stage) were fixed in 4% PFA for 24 h, and subsequently embedded in 15% sucrose, frozen, and sectioned into 20-μm thick sagittal sections. To block endogenous peroxidase activity, sections were treated with 0.5% H_2_O_2_ in 10 mM phosphate-buffered saline (PBS) containing 0.2% Triton X-100 (PBS-T) for 30 min. Subsequently, sections were blocked with 1% normal goat serum in PBS-T for 30 min. After washing with PBS-T, sections were incubated with primary antibody (Ab), rabbit polyclonal anti-DYX1C1 Ab (1:500, 14522-1-AP; Proteintech, Manchester, UK) or mouse monoclonal anti-reelin Ab (1:1,000, CR-50, a gift from Prof. Takaki Miyata, Nagoya University, Japan) at 4 °C for 24 h. After washing, the slices were incubated with goat anti-mouse IgG (1:250, BA-9200; Vector Laboratories, Burlingame, CA) or goat anti-rabbit IgG (1:500, BA-1000; Vector Laboratories) biotinylated secondary Ab at 25 °C for 2 h. Sections were then rinsed with PBS-T, and exposed to an avidin–biotin–horseradish peroxidase complex (1:1,000, ABC kit Vectastain PK-6100; Vector Laboratories) at 25 °C for 2 h. Visualization of the immunoreaction was achieved using nickel, diaminobenzidine, and H_2_O_2_, followed by microscopic examination. All slides were analyzed using the cellSens software imaging system (Olympus BX53, Tokyo, Japan) with a microscope (Olympus).

### Quantitative analysis of DYX1C1-positive cells

For quantitative analysis of DYX1C1-positive cell density, five different fields (100 μm in width) were captured from the cerebral cortex of each embryo (*n* = 7 embryos per developmental stage) using a light microscope (Olympus BX53) equipped with cellSens software (Olympus). The primitive cortical zone (PCZ), the outermost layer of the developing cortex, was defined based on neuroD2-positive regions at each developmental stage.^22^ The cortical plate (CP) was defined by its characteristic high cell density and radial organization of cells, while the ventricular zone (VZ) was identified by its pseudostratified epithelial structure.^23^ At later developmental stages (E17.5 and E20.5), the VZ region included the subplate (SP) and intermediate zone (IZ) due to developmental progression. Signal intensity of DAB immunostaining was analyzed using Fiji software.^24^ Cells showing a distinct DAB signal clearly distinguishable from the background with traceable borders were counted as positive cells. The population of DYX1C1-positive cells was calculated by dividing the number of positive cells by the measured area (width × height of each region).

### Fluorescence immunohistochemistry

The sections were processed for immunohistochemistry as previously described. Primary antibodies for fluorescence immunostaining, including mouse monoclonal anti-reelin Ab (1:1,000, CR-50) for identification of CR cells, mouse monoclonal anti-doublecortin (DCx) Ab (1:100, sc-271390; Santa Cruz Biotechnology, Santa Cruz, CA), mouse monoclonal anti-β3 tubulin Ab (1:100, Tuj1, sc-51670; Santa Cruz Biotechnology) for identification of neurons during development, mouse monoclonal anti-neuroD2 Ab (1:200, sc-365896; Santa Cruz Biotechnology) for detection of terminally differentiating neurons, mouse monoclonal anti-nestin Ab (1:1,000, ab22035; Abcam, Cambridge, UK) for identification of radial glial cells, and mouse monoclonal anti-ADP-ribosylation factor-like protein 13B (Arl13b) (1:1,000, 75-287; Antibodies Inc., Davis, CA) for visualization of primary cilia, were incubated with sections overnight at 4 °C. The specificity of anti-DYX1C1 antibody was confirmed by several control experiments. It was raised against a unique region of the DYX1C1 protein that has no significant homology to other TPR domain-containing proteins, thus minimizing potential cross-reactivity. This specificity was further validated by pre-absorption testing. The sections were then incubated with secondary antibodies, goat anti-rabbit IgG Alexa Fluor 488 Ab (1:500; A-11034, Invitrogen, Eugene, OR), and goat anti-mouse IgG Alexa Fluor 594 Ab (1:500; A-11032, Invitrogen) at 25 °C for 2 h. After washing with PBS-T, the slices were counterstained with 10 µg/ml Hoechst 33342. The sections were analyzed using a fluorescence microscope (Olympus BX53) and cellSens software imaging system (Olympus). Mercury lamps with 360–370 nm, 470–495 nm, and 530–550 nm bandpass filters were used to excite Hoechst 33342, Alexa Fluor 488, and Alexa Fluor 594, respectively. Emissions from Hoechst 33342, Alexa Fluor 488, and Alexa Fluor 594 were collected using a 420 nm long-pass filter, 510–550 nm band-pass filter, and 590 nm long-pass filter, respectively. All immunofluorescence staining was then examined using a confocal microscope, and co-localization was confirmed by sequential scanning, as follows.

### Confocal fluorescence microscopy

Confocal fluorescence imaging was performed using a Zeiss LSM 780 confocal microscope (Carl Zeiss Microscopy GmbH, Jena, Germany). Sections were prepared as previously described for fluorescence immunohistochemistry. The confocal laser scanning microscope was equipped with laser lines at 405, 488, and 561 nm excitation. Light emitted from Hoechst 33342, Alexa Fluor 488, and Alexa Fluor 594 was collected through a 420–470 nm, 500–550 nm, and 570–640 nm bandpass filter, respectively. Images were acquired with an optimal step size using a 63x oil immersion objective lens. The image acquisition settings were kept consistent for all comparison samples in the experiment. The acquired confocal images were processed and analyzed using the ZEN 2012 software (Carl Zeiss Microscopy GmbH). For three-dimensional (3D) reconstruction, z-stack images were processed using the 3D module of the ZEN software.

### RNA extraction and quantitative real-time PCR

Total RNA was purified from embryonic rat cerebral cortex according to the manufacturer’s instructions (Invitrogen, Carlsbad, CA). The quantitative real-time PCR was performed with 10 ng of total RNA using the One Step TB Green PrimeScript RT-PCR Kit II (Takara Bio, Tokyo, Japan). The following primers against DYX1C1 and glyceraldehyde-3-phosphate dehydrogenase (GAPDH) were used: 5’-CCAGAGGAAGGAGAAACCGC-3’ (DYX1C1-forward) and 5’-GCTTGTTTATGCAGCCACTCTT-3’ (DYX1C1-reverse), 5’-ACCACAGTCCATGCCATCAC-3’ (GAPDH-forward) and 5’-TCCACCACCCTGTTGCTGTA-3’ (GAPDH-reverse). The primers were designed using primer BLAST with a total length of 18–22 bp, Tm of 58–62 °C and GC% of 40–60. GAPDH was used as an internal reference control to normalize DYX1C1 expression levels. The thermocycling conditions were as follows: denaturation for 30 s at 95 °C followed by 40 cycles of 5 s at 94 °C, and 30 s at 60 °C. The reactions were amplified using the Real-time PCR StepOnePlus (Applied Biosystems, Waltham, MA). Expression levels were compared to E13.5, and relative expression levels were expressed as 2-ΔΔCt.^25^

### In situ hybridization histochemistry

Non-radioactive in situ hybridization was performed. RT-PCR products obtained with the primer set used for RT-PCR were cloned into pCRII vector (TA Cloning® Kit Dual Promoter (pCR®II), Invitrogen) after gel purification. An insertion check was performed based on the PCR product size using the M13 forward primer site in pCRII vector and the DYX1C1 primer pair. Sense and antisense probes for DYX1C1 were prepared via in vitro transcription using plasmids. Probes were synthesized using digoxigenin-11UTP (Roche Diagnostics, Manheim, Germany) using in vitro transcription system (Riboprobe® System, Promega, Madison, WI) after linearization of the template with *XbaI* (for sense probe, Takara Bio) or *BamHI* (for antisense probe, Takara Bio) prior to RNA synthesis using T7 or SP6 RNA polymerase. The sections were hybridized for 18 h at 37 °C with the antisense probes in the hybridization buffer including 10 mM dithiothreitol, 50% formamide, 5× SSC (1× SSC; 0.15 M NaCl, 0.015 M sodium citrate, pH 7.0), 5× Denhardt’s solution, 200 µg/ml yeast tRNA, and 10% dextran sulfate, under Parafilm coverslips in a humid chamber. The sections were washed in 4×, 2×, 1× and 0.5× SSC for 5 min each at room temperature. Non-specific hybridization (negative control) was determined by incubation with the sense probes. Signals were generated using alkaline phosphatase-labeled anti-digoxigenin Fab fragments (Roche Diagnostics) and added to the chromogenic substrate nitroblue tetrazolium chloride/ 5-brom-4-chloro-3-indolyl phosphate (Roche Diagnostics) for visualization.

### Measurement of cilia length

Cilia length was measured in DYX1C1-positive and negative cells in the developing cortex at E15.5 of 12 embryos. Embryonic rat brain sections were processed for double immunofluorescence staining as described above, using antibodies against DYX1C1 and the ciliary marker Arl13b. All slides were analyzed using the cellSens software imaging system (Olympus) with a microscope (Olympus). Primary cilia were identified using Arl13b immunostaining. The lengths of the Arl13b-positive cilia were measured manually using the cellSens measurement function by tracing a segmented line from the cilia base at the centriole to the tip. At least 50 cilia were measured in the DYX1C1-positive or the DYX1C1-negative cells. Only the cells within the cortical plate were included in the analysis.

### Statistical analysis

The statistical analyses were conducted using EZR (Easy R) version 1.60 (Saitama Medical Center, Jichi Medical University, Saitama, Japan) which is a graphical user interface for R (The R Foundation for Statistical Computing, Vienna, Austria; https://www.r-project.org/).^26^ The data representation was meticulously selected to effectively convey both the variability inherent in our measurements and the precision of our estimated means. The data are presented as mean ± standard deviation (SD). A *p*-value of less than 0.05 was considered statistically significant. The following levels of statistical significance were employed: *p < 0.05, **p < 0.01, and ***p < 0.001. Because the prior Bartlett’s test detected heterogeneity of variances, Welch’s one-way analysis of variance (ANOVA) was conducted, followed by Games-Howell *post hoc* test for multiple comparisons, to analyze temporal changes in DYX1C1-positive cell population in each cortical area (PCZ, CP, or VZ) across four developmental stage in the immunohistochemistry (E13.5, E15.5, E17.5 and E20.5; *n* = 7 per developmental stage, 5 fields each, total of 35 fields per stage). After confirming the homogeneity of variances using Bartlett’s test, one-way ANOVA was performed, followed by Tukey’s *post hoc* test, to analyze temporal changes in DYX1C1 mRNA expression across developmental stages. For the comparison of cilia length between DYX1C1-positive (*n* = 71) and DYX1C1-negative cells (*n* = 61), the unpaired *t*-test was employed.

## Results

### Localization of DYX1C1-positive cells in the developing cortex

**Fig. 1:**
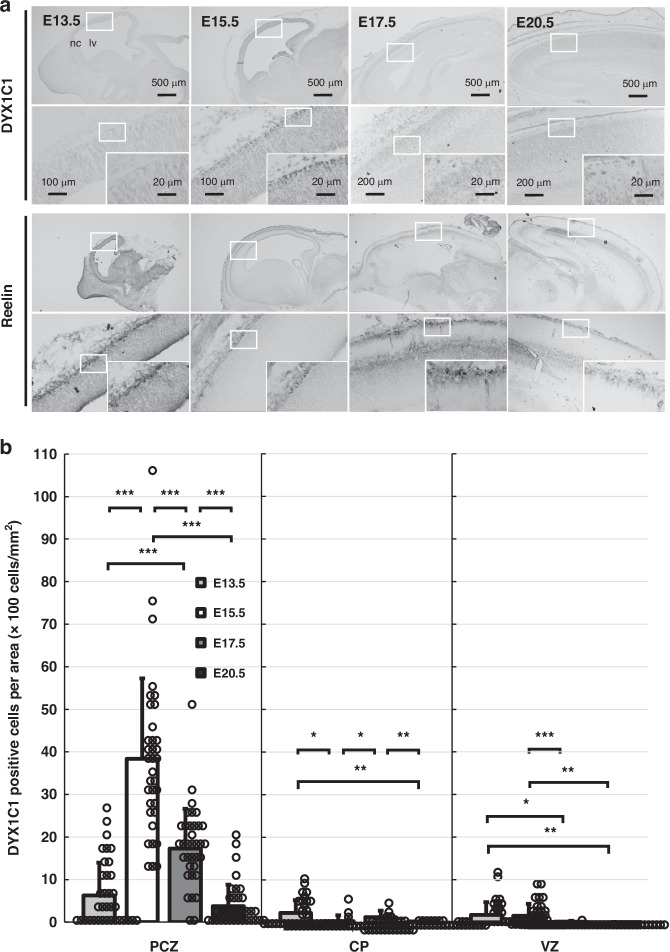
Localization of DYX1C1 and reelin proteins during cerebral cortex development in rats. **a** Representative immunohistochemical staining shows the spatiotemporal distribution of DYX1C1 and reelin at different embryonic stages (E13.5–E20.5, *n* = 7 embryos per stage). DYX1C1-positive cells are located in the outer CP, peaking at E15.5, whereas reelin-positive CR cells are located in the MZ. Insets show higher magnification views. Scale bar = 500 μm (upper panels), 100 μm (lower panels of E13.5 and E15.5), 200 μm (lower panels of E17.5 and E20.5), 20 μm (insets). **b** Quantitative analysis for the population of DYX1C1-positive cells in the developing cerebral cortex. The analysis was conducted on five distinct fields, each measuring 100 µm in width, from each embryo. The data are presented as the means ± SD (bars) with individual data points shown as dots (*n* = 7 embryos per stage, 5 fields each). The values represent the number of positive cells per unit area, expressed in cells/mm^2^. At E17.5 and E20.5, the VZ region encompasses the subplate (SP) and intermediate zone (IZ) as a consequence of developmental progression. *p < 0.05, **p < 0.01, ***p < 0.001 (Bartlett’s test *p* < 0.001, Welch’s one-way ANOVA, followed by Games-Howell *post-hoc* test). CP cortical plate, lv lateral ventricle, MZ marginal zone, nc neocortex, PCZ primitive cortical zone, pia pia mater, VZ ventricular zone.

Immunohistochemistry was performed to investigate the spatiotemporal distribution of DYX1C1 in developing rat cerebral cortex (Fig. 1). Representative images from more than four embryos at each developmental stage are presented. Immunohistochemistry revealed distinct spatiotemporal distribution patterns of DYX1C1-positive cells in the developing cerebral cortex of rats (Fig. 1a, upper panels). The distribution of DYX1C1 in the developing cortex differed according to fetal age, peaking at E15.5. At E15.5, DYX1C1 staining was stronger in cells located in the layer close to the pial surface of the cortex than that in the other layers.

It was not clear whether the DYX1C1-positive cells were in the marginal zone (MZ) or the outermost layers of the CP. To elucidate the distribution of DYX1C1-positive cells relative to reelin-expressing CR cells, immunohistochemical analysis was performed using the CR-50 antibody for reelin (Fig. 1a, lower panels). While reelin-positive CR cells were localized in the MZ, DYX1C1-positive cells appeared to be positioned slightly ventricular to the MZ. Morphological differences between DYX1C1-positive cells and reelin-positive CR cells were observed, with CR cells exhibiting horizontal extension. Based on their characteristic position and morphology near the pial surface, these intensely labeled cells appeared to be located in the PCZ, the outermost layer of the developing cortex.

The analysis of DYX1C1-positive cell population revealed the existence of distinct temporal patterns across different cortical regions (Fig. 1b). Prior to statistical analysis, Bartlett’s test revealed significant heterogeneity of variances in all regions (PCZ: χ² = 63.2, CP: χ² = 115.0, VZ: χ² = 304.4; all df = 3, *p* < 0.001), then a Welch’s one-way ANOVA and Games-Howell *post-hoc* test were employed to analyze significance of temporal changes in each cortical region. In the PCZ, there were statistically significant differences in the DYX1C1-positive cell population across the various developmental stages (*p* = 4.26 × 10^−17^). The DYX1C1-positive cells showed a notable increase from E13.5 (629 ± 766 cells/mm²) to E15.5 (3779 ± 1900 cells/mm²; *p* = 5.82 × 10^−11^ vs. E13.5), followed by a significant decline at E17.5 (1729 ± 934 cells/mm²; *p* = 3.48 × 10^−6^ vs. E15.5), and a further reduction at E20.5 (371 ± 509 cells/mm²; *p* = 8.01 × 10^−12^ vs. E15.5). In the CP, the temporal changes of DYX1C1-positive cells were more subtle but significant across stages (*p* = 7.16 × 10^−5^). The highest population was observed at E13.5 (230 ± 329 cells/mm²), with a subsequent gradual decline throughout the developmental period to E20.5 (30 ± 39 cells/mm²; *p* = 0.006 vs. E13.5). The DYX1C1-positive cell population in the VZ demonstrated notable temporal alterations (*p* = 6.46 × 10^−6^), with the highest population observed at E15.5 (213 ± 282 cells/mm²), although the population decreased at later stages (E17.5: 11 ± 23; E20.5: 3 ± 10 cells/mm²). These findings demonstrate that DYX1C1 expression is most prominently regulated in the PCZ during cortical development, exhibiting a distinct peak at E15.5 that is significantly higher than the expression levels observed in other cortical regions or at other developmental stages.

### Lack of co-localization between DYX1C1 and reelin

**Fig. 2:**
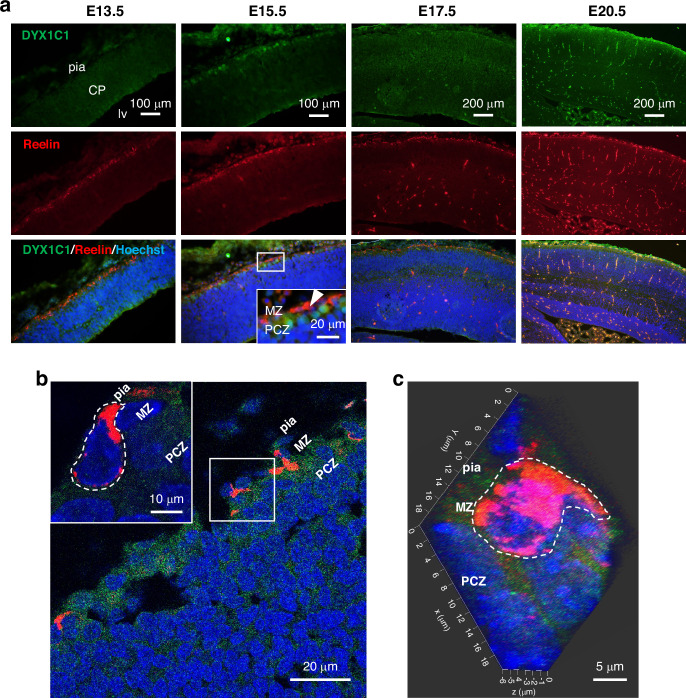
Localization of DYX1C1 and reelin-positive cells in the developing cerebral cortex. **a** Representative images of double immunohistochemistry, showing that DYX1C1 (green) and reelin (red) have distinct but adjacent localization patterns at E15.5 (*n* = 9 embryos per stage). DYX1C1-positive cells located just ventral to the reelin-positive CR cell layer. Scale bar = 100 μm (main panels of E13.5 and E15.5), =200 μm (main panels of E17.5 and E20.5), =20 μm (insets). **b** High-resolution confocal microscopy images of DYX1C1 and reelin. Scale bar = 20 μm (main panels), =10 μm (insets). **c** 3D reconstruction of confocal z-stack images of DYX1C1 and reelin-positive CR cells. Scale bar = 20 μm. The representative reelin-positive CR cells are circled by white dashed lines. CP cortical plate, lv lateral ventricle, MZ marginal zone, nc neocortex, PCZ primitive cortical zone, pia pia mater.

To confirm the precise anatomical localization of DYX1C1-positive cells relative to the MZ, we performed double immunofluorescence staining for DYX1C1 and reelin (Fig. 2). DYX1C1-positive cells were distinct from CR cells but closely adjacent to the CR cells and were particularly prominent at E15.5, suggesting the localization of DYX1C1 in the PCZ (Fig. 2a). Analysis with the Zeiss LSM 780 confocal microscope further confirmed this mutually exclusive localization pattern of DYX1C1 and reelin-positive CR cells, with DYX1C1-positive cells consistently located just below the MZ (Fig. 2b). The 3D reconstruction of confocal z-stack images provided further validation of this distinct spatial organization, demonstrating consistent segregation of DYX1C1 and reelin expression domains throughout the tissue depth (Fig. 2c).

### DYX1C1 mRNA expression profiles during development

**Fig. 3:**
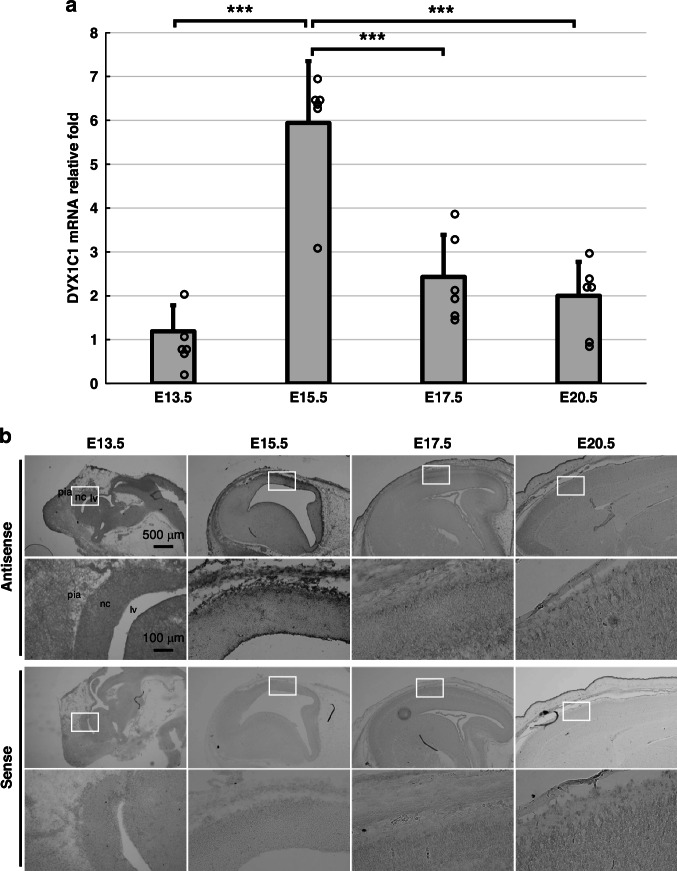
Temporal DYX1C1 mRNA expression in the cerebral cortex during development. **a** Quantitative real-time PCR analysis of DYX1C1 mRNA levels at embryonic stages E13.5–E20.5. The data are presented as means ± SD (bars) with individual data points shown as dots (*n* = 6 per stage). ***p < 0.001 between indicated groups (Bartlett’s test *p* = 0.347, one-way ANOVA, followed by Tukey’s *post-hoc* test). **b** Representative images of in situ hybridization for DYX1C1 mRNA in the cortex at E13.5-E20.5 using antisense probes or control sense probes (11–15 embryos/stage). Each lower panel shows higher magnification image of white boxed area in the upper panel. nc neocortex, lv lateral ventricle, pia pia mater.

Temporal changes in DYX1C1 mRNA expression in the cerebral cortex during rat cortical development were investigated using quantitative real-time PCR (Fig. 3a). DYX1C1 mRNA expression increased rapidly from E13.5 to E15.5 and then gradually decreased. After confirming homogeneity of variances by Bartlett’s test (*p* = 0.347), one-way ANOVA revealed significant differences in expression levels across different developmental stages (*p* = 3.10 × 10^−7^). A Tukey’s *post hoc* analysis revealed that DYX1C1 mRNA expression levels at E15.5 (5.94 ± 1.38) were markedly elevated in comparison to those observed at E13.5 (1.19 ± 0.60; *p* = 4.00 × 10^−7^), E17 (2.43 ± 0.98; *p* = 2.90 × 10^−5^), and E20.5 (2.00 ± 0.83; *p* = 5.80 × 10^−6^). No significant differences in expression levels were observed between E13.5 and E17.5 (*p* = 0.166), E13.5 and E20.5 (*p* = 0.507), or E17.5 and E20.5 (*p* = 0.872). Spatiotemporal expression analysis using in situ hybridization histochemistry corroborated these findings, showing the strongest signal in the PCZ with peak expression observed at E15.5 (Fig. 3b).

### Co-localization of DYX1C1 with neuronal developmental markers

**Fig. 4:**
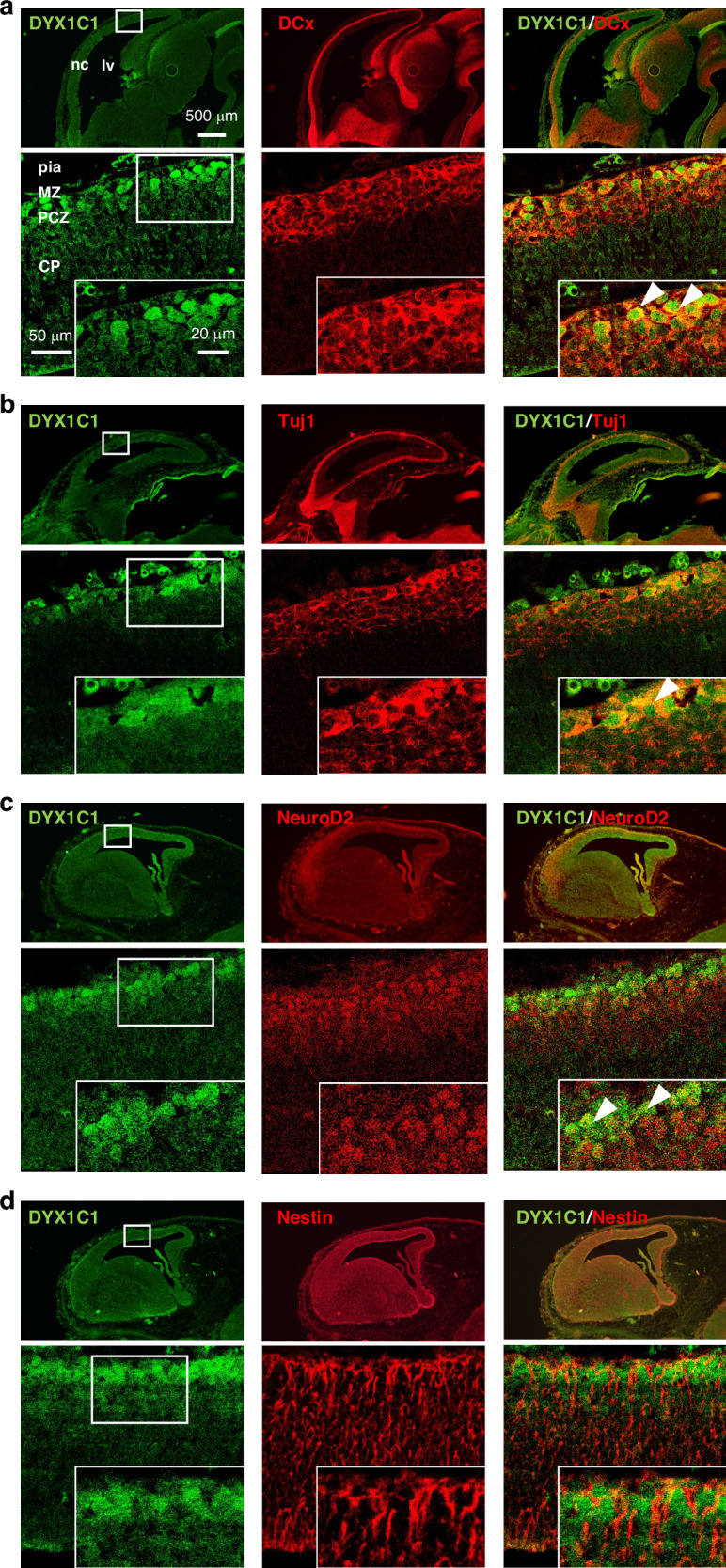
DYX1C1 and neuronal developmental markers in the cerebral cortex at E15.5. **a** Representative images of double immunostaining of DYX1C1 (green) and a neuronal marker expressed during development DCx (red). Arrowheads in the high magnification inset show DYX1C1- and DCx-double positive cells. **b** DYX1C1 (green) and a neuronal marker during development, β3-tubulin (Tuj1, red). Arrowhead shows DYX1C1-positive cells co-expressing Tuj1. **c** DYX1C1 (green) and a neuronal differentiation factor neuroD2 (red) expression. Arrowheads show DYX1C1 and neuroD2-double positive cells. **d** Double immunostaining of DYX1C1 (green) and a radial glial marker nestin (red). (**a**–**d** *n* = 6 ~ 8 embryos/stage) The lower panels display high-resolution images taken by a Zeiss LSM 780 confocal microscope, facilitating visualization of protein co-localization patterns. Scale bar = 500 μm (upper panels), 50 μm (lower panels), 20 μm (insets). nc neocortex, lv lateral ventricle, pia pia mater, CP cortical plate, MZ marginal zone, PCZ primitive cortical zone.

To examine the stage of neuronal differentiation in DYX1C1-positive cells, we performed double immunostaining for neuronal developmental markers at E15.5. A co-localization analysis was conducted using high-resolution confocal microscope with sequential scanning. DYX1C1-positive cells in the outer layers of CP were co-stained with neuronal marker DCx and β3 tubulin (Tuj1) which are expressed during neuronal development, and neuronal differentiation factor neuroD2 (Fig. 4a–c). NeuroD2-positive cells also showed a population of cells with intranuclear signals in the outer layers of CP, PCZ (Fig. 4c). Furthermore, co-staining of DYX1C1 with nestin, a marker of radial glia, showed no co-localization (Fig. 4d). Each lower panel was captured using a confocal microscope for detailed visualization of co-localization patterns.

### Cilia on the DYX1C1-expressing cells

**Fig. 5:**
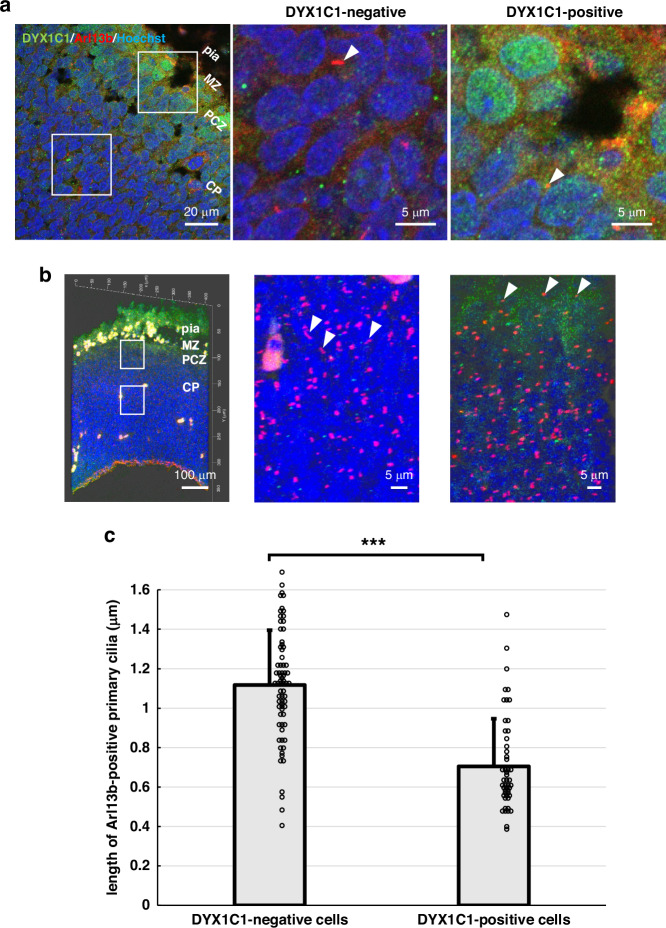
Primary cilia characteristics in DYX1C1-expressing cells in the embryonic rat cerebral cortex at E15.5. All images were acquired using a Zeiss LSM 780 confocal microscope. **a** Immunofluorescence staining of DYX1C1 (green), the ciliary marker Arl13b (red), and counter nuclear staining with Hoechst dye (blue). Left panel: overview of the cortical region at low magnification. Middle and right panels: high magnification images of DYX1C1-negative and DYX1C1-positive regions. **b** Z-stack 3D reconstructed confocal images of DYX1C1 and primary cilia in the cerebrocortical region. Left panel: lower magnification image. Middle and right panels: higher magnification images in DYX1C1-negative and DYX1C1-positive regions. **c** Quantification of the length of Arl13b-positive primary cilia in DYX1C1-negative vs. DYX1C1-positive cells within the cortical plate at E15.5 of 12 rat embryos. Data are presented as means ± SD (bars) with individual measurements shown as dots. Each dot represents a single primary cilium measurement (*n* = 71 and 61 cilia for DYX1C1-negative and DYX1C1-positive cells, respectively). ***p < 0.001 (unpaired *t*-test). White arrowheads indicate primary cilia in each region. CP cortical plate, MZ marginal zone, nc neocortex, PCZ primitive cortical zone, pia pia mater.

To examine the characterization of primary cilia on DYX1C1-positive cells, co-staining with Arl13b was performed at E15.5 (Fig. 5). Immunostaining using Anti-Arl13b antibody visualized primary cilia (Fig. 5a, b). 3D reconstructed confocal images of DYX1C1 clearly showed primary cilia in the cerebrocortical region (Fig. 5b). DYX1C1-positive cells which were mainly seen in the PCZ appeared to have shorter primary cilia than DYX1C1-negative cells (Fig. 5a). This trend is confirmed by 3D reconstructed confocal images of DYX1C1 and primary cilia (Fig. 5b). Therefore, the length of Arl13b-immunopositive primary cilia in both DYX1C1-positive cells and DYX1C1-negative cells was measured, and shown in Fig. 5c. The quantitative analysis demonstrated that DYX1C1-positive cells possessed significantly shorter primary cilia compared to DYX1C1-negative cells. The average cilia length in DYX1C1-positive cells was 0.70 ± 0.24 μm (*n* = 61), while DYX1C1-negative cells exhibited longer cilia with an average length of 1.12 ± 0.28 μm (*n* = 71) (*p* = 6.76 × 10^−14^), showing approximately 37% reduction in DYX1C1-positive cells.

## Discussion

Our study aimed to investigate the spatiotemporal dynamics of DYX1C1 expression during cortical development, based on previous research that identified DYX1C1 expression in various brain regions, including the neocortex, hippocampus, and choroid plexus.^17^ Our study revealed a specific temporal pattern of DYX1C1 expression peaking at E15.5, which differs from in situ hybridization in the previous study that reported peak expression at round E17.^17^ We utilized Sprague-Dawley rats rather than Wistar rats used in the previous study.^17^ The strain-specific differences in neurodevelopmental timing and gene expression patterns may contribute to the temporal shift in peak expression we observed. We revealed robust expression of DYX1C1 protein and mRNA, primarily within the outer layers of the CP, with a notable peak observed at E15.5. The number of DYX1C1-positive cells was significantly higher in the PCZ in comparison to other CP regions. The uppermost layer of the CP is designated as PCZ.^22^ In the PCZ, the dynamic mode change of neuronal migration for lamination occurs in an inside-out manner, progressing from “locomoting” to “terminal translocation“.^27^ Terminal translocation, which is the final mode of migration, plays a pivotal role in the cellular positioning of neurons. This specific spatial localization suggests a potential role for DYX1C1 in shaping cerebrocortical architecture during critical stages of development.

Our analysis revealed distinct spatial organization between DYX1C1-positive and CR cells in the developing cerebral cortex. While reelin-positive CR cells were in the MZ, DYX1C1-positive cells were in the PCZ. This precise spatial segregation, without overlapping populations of CR cells and DYX1C1-positive cells. The exclusive localization pattern of these cell populations implies a potential regulatory relationship: DYX1C1-positive cells may be positioned to respond to signals from reelin-producing CR cells, rather than producing reelin themselves. This spatial arrangement could be crucial for the proper establishment of cortical layers, as DYX1C1-expressing cells places in an optimal position to respond to reelin signaling during critical periods of cerebrocortical development. These findings are supported by the gene ontology analysis using the Database for Annotation, Visualization, and Integrated Discovery (DAVID), which implied an association between the DYX1C1 gene network and CR cells.^28^ CR cells regulate the migration of neurons and the formation of cortical layers through the secretion of the extracellular glycoprotein reelin.^29^ Although reelin plays a crucial role in neuronal migration and layer formation,^30^ our study suggests that DYX1C1 is also involved in cortical organization, possibly influencing neuronal migration and the development of cerebrocortical layers. Reelin is secreted by CR cells in the MZ during brain development and is involved in the migration and formation of layers in the cerebral cortex, cerebellar cortex, and hippocampus.^31^ This indicates that DYX1C1 is a pivotal factor in reelin-mediated processes in the neocortex and hippocampus. Short-term memory deficits have been observed in cases of DYX1C1 translocations or variants, and the results were reproducible.^8,32^ Previous reports have indicated that the repression of DYX1C1 expression via short hairpin RNA (shRNA) leads to malformations in the cerebral cortex and hippocampus.^16–18,33,34^ These associations underscore the multifaceted roles of DYX1C1 in neurodevelopmental processes and its potential as a therapeutic target in dyslexia and related disorders. This spatiotemporal expression of DYX1C1 opens avenues for further investigation of the molecular mechanisms underlying cerebrocortical patterning.

Our immunohistochemical analysis demonstrated that DYX1C1 expression was present not only in the outer CP but also in cells within the VZ at E15.5. Taken together with previous studies showing that DYX1C1 knockdown disrupts neuronal migration to cause subcortical heterotopias,^16–18^ the present results indicating DYX1C1 expression in the outermost layer of the CP at 15.5 suggest an involvement of DYX1C1 throughout neuronal migration stage, from initial migration to terminal translocation.

Moreover, our study highlights an intricate network of interactions involving DYX1C1, including its reported association with DCDC2 and microtubule proteins.^35^ These interactions suggest a potential role for DYX1C1 in modulating neuronal migration processes, which could affect cortical layer formation and organization. *In utero* knockdown of DYX1C1 and DCDC2 using RNAi results in an “over-migration” phenotype, whereby transfected neurons migrate beyond their assumed laminar location.^18,36^ DYX1C1 is associated with microtubule proteins, whereas DCDC2 is involved in microtubule stabilization.^28,37^ Our findings contribute to the growing understanding of the molecular mechanisms governing cortical development and provide insights into the potential implications of DYX1C1 dysfunction in neurodevelopmental disorders such as dyslexia.

We also observed the co-localization of DYX1C1 with neuroD2, a transcription factor involved in terminal translocation during cortical development,^38^ with the notable exception of CR cells.^39^ This suggests that DYX1C1 may be involved in the final stage of terminal translocation, which is consistent with the fact that neuroD2 is predominantly expressed in the cortex during peak terminal translocation.^40^ This idea is supported by a previous study in which DYX1C1 was among the set of genes targeted by neuroD2.^41^ The expression profile of DYX1C1 coincides with peak terminal translocation, further supporting its potential role in this process.^41^ Interestingly, reelin triggers the detachment of radial glia and induces a switch in the migration mode from locomotion to terminal translocation.^42^

DYX1C1 plays a role not only in DD, neuronal migration, but also in primary cilia. DYX1C1 binds to the basal body in primary cilia.^28,43^ DYX1C1 and DCDC2 proteins have been shown to interact with centrosomal P4.1-associated protein (CPAP).^35^ Arl13b is a member of the regulatory GTPase family and is highly enriched in the ciliary membrane^44,45^; gene mutation in Arl3b can cause Joubert Syndrome.^46^ Our investigation revealed an unexpected and intriguing finding regarding primary cilia in DYX1C1-expressing cells. Although DYX1C1 is known to be expressed in primary cilia and to associate with the basal body, our observations revealed that DYX1C1-positive cells possessed significantly shorter primary cilia than DYX1C1-negative cells (0.70 μm vs 1.12 μm, *p* < 0.001). This considerable discrepancy in cilia length may be indicative of functional alterations in the primary cilium as cells undergo their migratory processes under the influence of reelin signaling. The DYX1C1 protein may change the conformation upon reaching the CR cell layer, either binding to or dissociating from the primary cilium complex. Such conformational changes may affect ciliary length and enhance DYX1C1 detection by immunohistochemistry. This tentative interpretation suggests a link between reelin signaling, DYX1C1 function, and ciliary dynamics during neuronal positioning. Primary cilia detect molecular cues that direct neuronal migration. The reduction in cilia length in DYX1C1-positive cells may indicate a transition in cellular behavior, marking the completion of migration and the initiation of terminal translocation. This is consistent with DYX1C1 expression in the outer cerebrocortical layers and its co-localization with neuroD2. During the migration of neurons in the cerebral cortex, the primary cilia act as antennas to search for polarity.^47^ This may indicate that DYX1C1 functions as a cytoskeletal protein in terminal translocation rather than as a ciliary protein during migration. DYX1C1, similar to DCDC2, contributes to microtubule stabilization and may play a role in terminal translocation as neurons settle into their proper position after neuronal migration. Although some changes in primary cilia length and other factors are known,^48^ few studies have examined and compared changes in primary cilia length in the developing cerebral cortex. Future studies may reveal whether the increase in DYX1C1 expression in settled-down neurons reduce the sensing function of cilia.

Our findings in rats shed light on DYX1C1’s role in cortical development, but caution is needed when translating them to human dyslexia. Animal models are useful to examine biological changes, but they are not suitable to evaluate unique human dyslexia.^6,49^ However, our findings in rats revealed natural spatiotemporal expression pattern of DYX1C1 in cortical development, and contribute to understanding the biological basis of dyslexia. Identifying specific temporal windows of DYX1C1 expression and its interaction with other developmental factors may inform future therapeutic strategies, particularly those aimed at early intervention.

In conclusion, this study provides three key findings to the field of cerebral cortex development. First, we have provided spatiotemporal expression patterns of endogenous DYX1C1 predominantly in the outermost layers of the cortical plate during cerebrocortical development in rats. Second, our study has elucidated the precise spatial relationship between DYX1C1-positive cells and reelin-expressing CR cells. Although DYX1C1-positive cells are distinct from CR cells, they are closely adjacent to the CR cells, and co-localize with neuronal markers expressed during cortical development, indicating its contribution to neuronal migration and layer formation. Third, DYX1C1-positive cells mainly in the PCZ possess shorter primary cilia than DYX1C1-negative cells, suggesting the completion of migration. Further exploration of the functional interactions of DYX1C1, particularly its relationship with reelin and its influence on hippocampal development and their downstream effects, holds promise for understanding the pathophysiology of dyslexia and related neurodevelopmental disorders, and for identifying therapeutic targets. Thus, our study contributes to current knowledge by providing comprehensive insights into the expression dynamics of DYX1C1 and its functional implications during cerebral cortex development. The identification of novel interactions and potential roles of DYX1C1 opens new avenues for research and therapeutic interventions in neurodevelopmental disorders.

## Data Availability

The datasets used and/or analyzed in the current study are available from the corresponding author upon reasonable request.
